# Orbital Apex Metastasis from Adenoid Cystic Carcinoma: Acute Loss of Vision and Subsequent Recovery with the Radiation

**DOI:** 10.7759/cureus.1869

**Published:** 2017-11-22

**Authors:** Lauren Crawford, Sanjeev Sharma

**Affiliations:** 1 Neurology, Indiana University School of Medicine; 2 Department of Radiation Oncology, St. Mary's Medical Center

**Keywords:** orbital metastasis, loss of vision, recovery of vision, compressive optic neuropathy, adenoid cystic carcinoma, intensity modulated radiation therapy

## Abstract

Orbital apex metastasis from adenoid cystic carcinoma (ACC) is rare. We present a patient with known metastatic ACC presented with a rapidly declining vision with visual acuity oculus dexter (OD) equal to counting fingers at two feet. On imaging, she was found to have a right orbital apex tumor causing compressive optic neuropathy. She received the intensity modulated radiation therapy (IMRT). After completion of the therapy, she had regained essentially a full vision with visual acuity OD of 20/30 without corrective lenses. The treatment rationale and pertinent literature are discussed in this article.

## Introduction and background

Visual loss due to extraocular orbital metastases is a relatively uncommon [1] and devastating complication of malignancy. The adenoid cystic carcinoma (ACC) is an uncommon malignancy which arises within secretory glands, most commonly of the head and neck [2]. It typically has a relatively indolent but relentless course [2]. In this review, we report the case of a 58-year-old female with the clinical history of cavernous sinus and right temporal lobe metastases, who presented with acute right visual loss due to compressive optic neuropathy. She received radiotherapy with subsequent recovery of vision. We also review the current literature regarding the treatment of ACC metastases with radiotherapy and the use of radiotherapy in orbital metastases.

## Review

Case presentation

**Figure 1:**
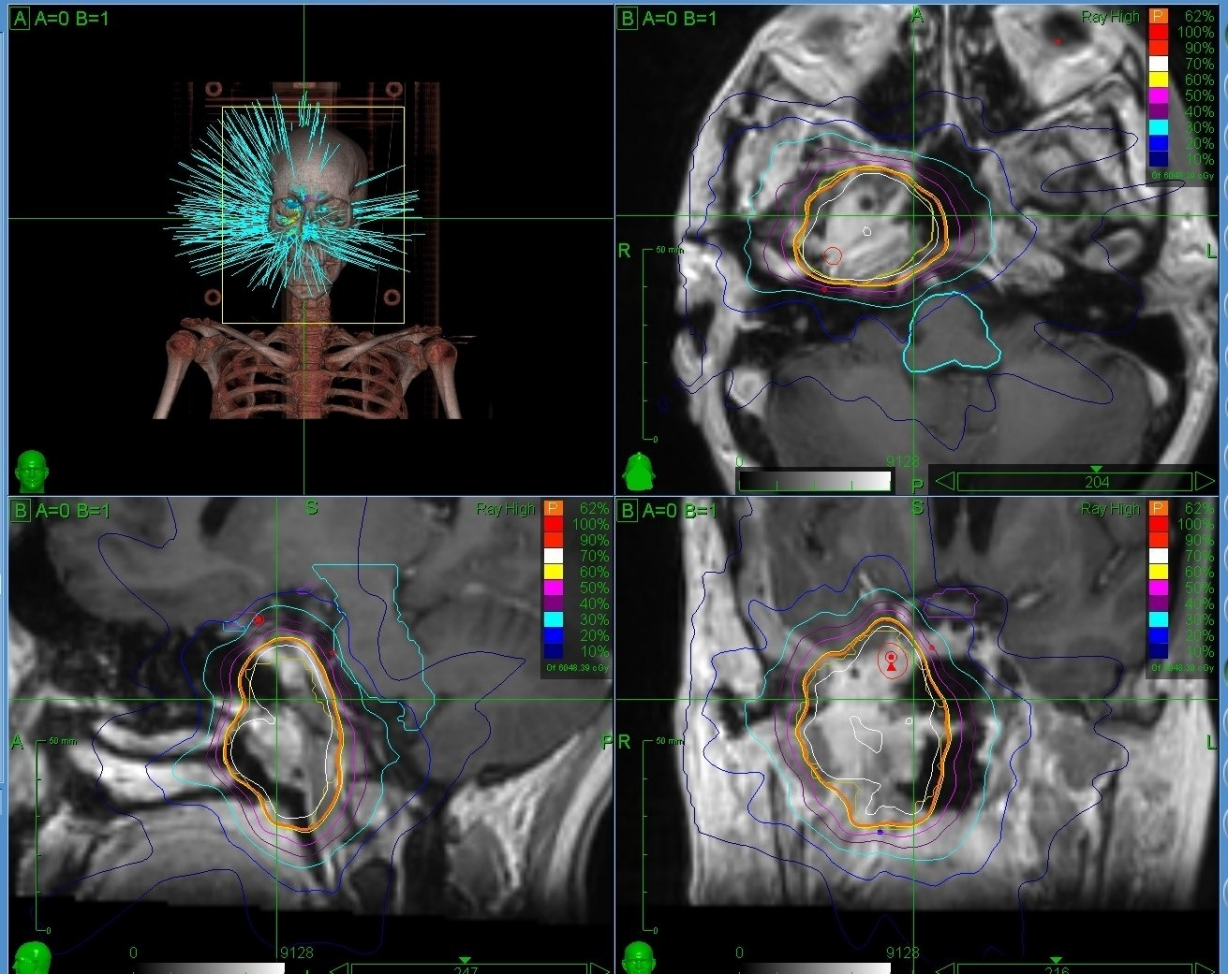
The stereotactic body radiation therapy treatment plan for the primary tumor site in the nasopharynx.

**Figure 2:**
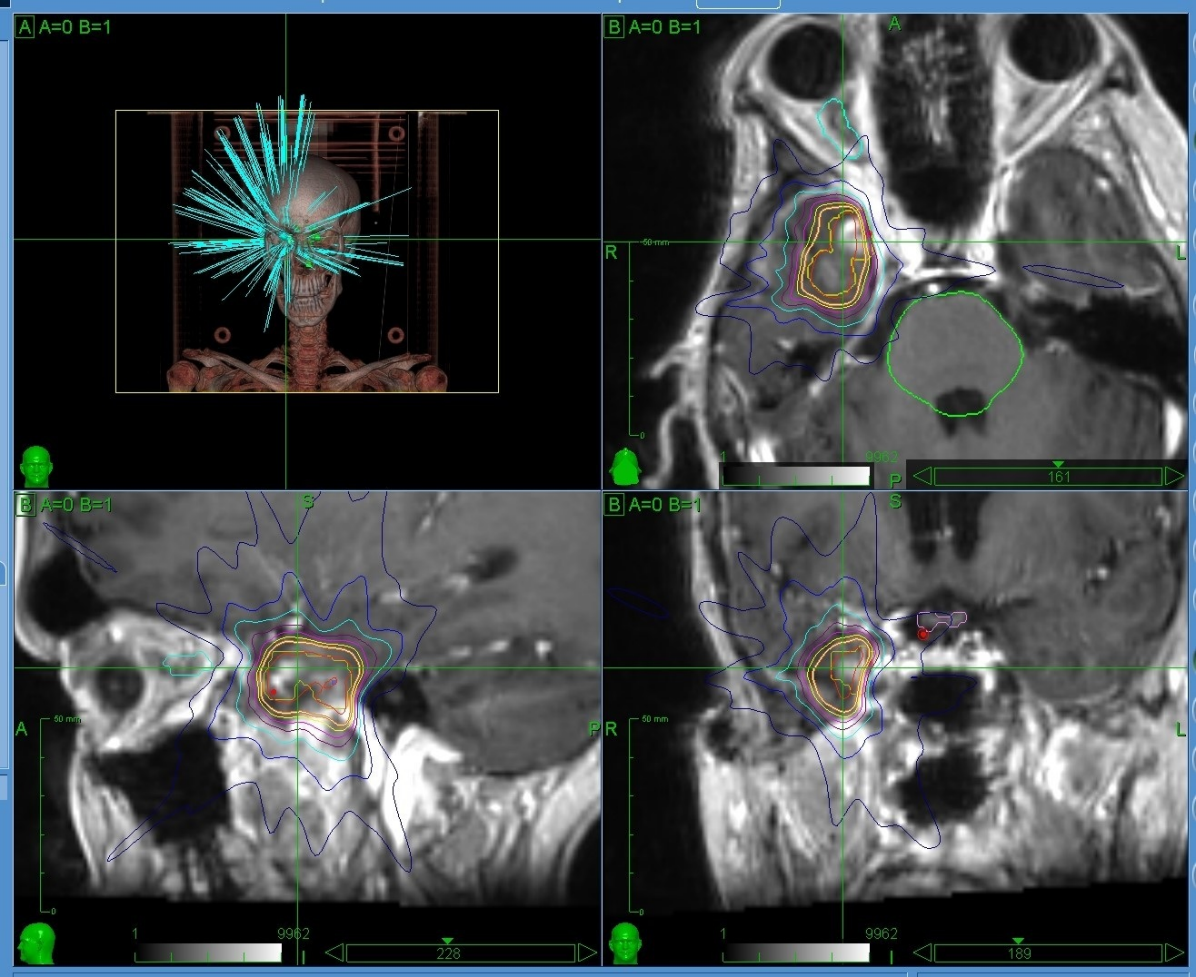
The hypofractionated radiotherapy treatment plan for new metastases.

A 58-year-old Caucasian female presented in April 2012 with new onset right parasthesias in the ophthalmic nerve (V1), the maxillary nerve (V2) and the mandibular nerve (V3) distributions with associated neuralgia. The magnetic resonance imaging (MRI) revealed a right cavernous sinus mass lesion. At an outside institution, the patient underwent partial resection. The pathology revealed ACC with perineural invasion. The staging positron emission tomography (PET) and biopsy revealed the site of primary in the right lateral wall of the nasopharynx. The patient was treated with the stereotactic body radiation therapy (SBRT) on Cyberknife ™ receiving 3750 centi-Gray (cGy) in five fractions of 750 cGy per fraction (Figure 1) to both areas of disease, completed the treatment in August 2012. Post-treatment, her pain abated, but the facial paresthesias persisted. Her vision was normal. The follow-up MRI in September 2014 revealed a new 4 mm area of enhancement in the medial right temporal lobe. This was clinically asymptomatic. The new area was followed by serial MRIs, which showed slow progression, evolving into two adjacent areas of enhancement. These two new areas were treated with the hypo-fractionated radiotherapy on Cyberknife® receiving 4000 cGy in 10 fractions of 400 cGy per fraction (Figure 2), completed in October 2015.

**Figure 3:**
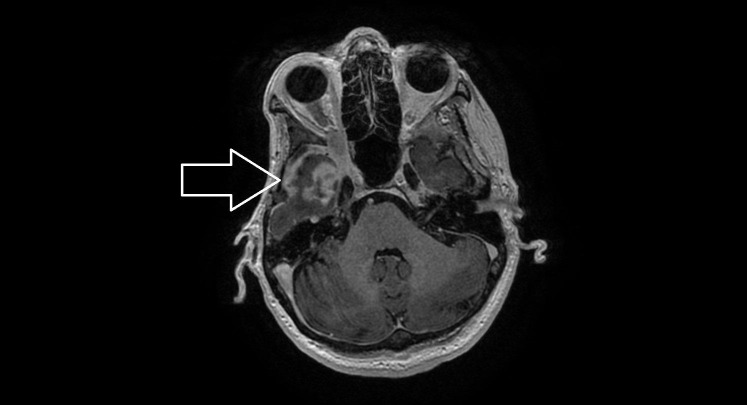
The magnetic resonance imaging of the orbital metastases causing compressive neuropathy.

In August 2016, the patient presented with blurred vision in the right eye, which was further assessed by the ophthalmology and felt to be secondary to cataracts. The follow-up MRI at that time was stable. She underwent cataract removal in September 2016. Following the surgery, the patient reported progressive right visual loss with orbital and retro-orbital pain. On examination during October 2016, the patient had a fixed right pupil with the blurred vision of the right eye. She was only able to discern the objects and was not able to read the written words. Due to the concern of possible radiation-induced optic neuropathy (RION), she was initiated on high dose methylprednisolone 1000 g IV QD X three days and pentoxifylline 400 mg TID with vitamin E 1000 IU daily as per publications for the treatment of RION [3]. After day two of intravenous (IV) steroids, the patient’s pain resolved and the right pupil became reactive. However, the vision remained unchanged. The MRI revealed a right orbital apex tumor consistent with metastasis causing compressive optic neuropathy (Figure 3).

**Figure 4:**
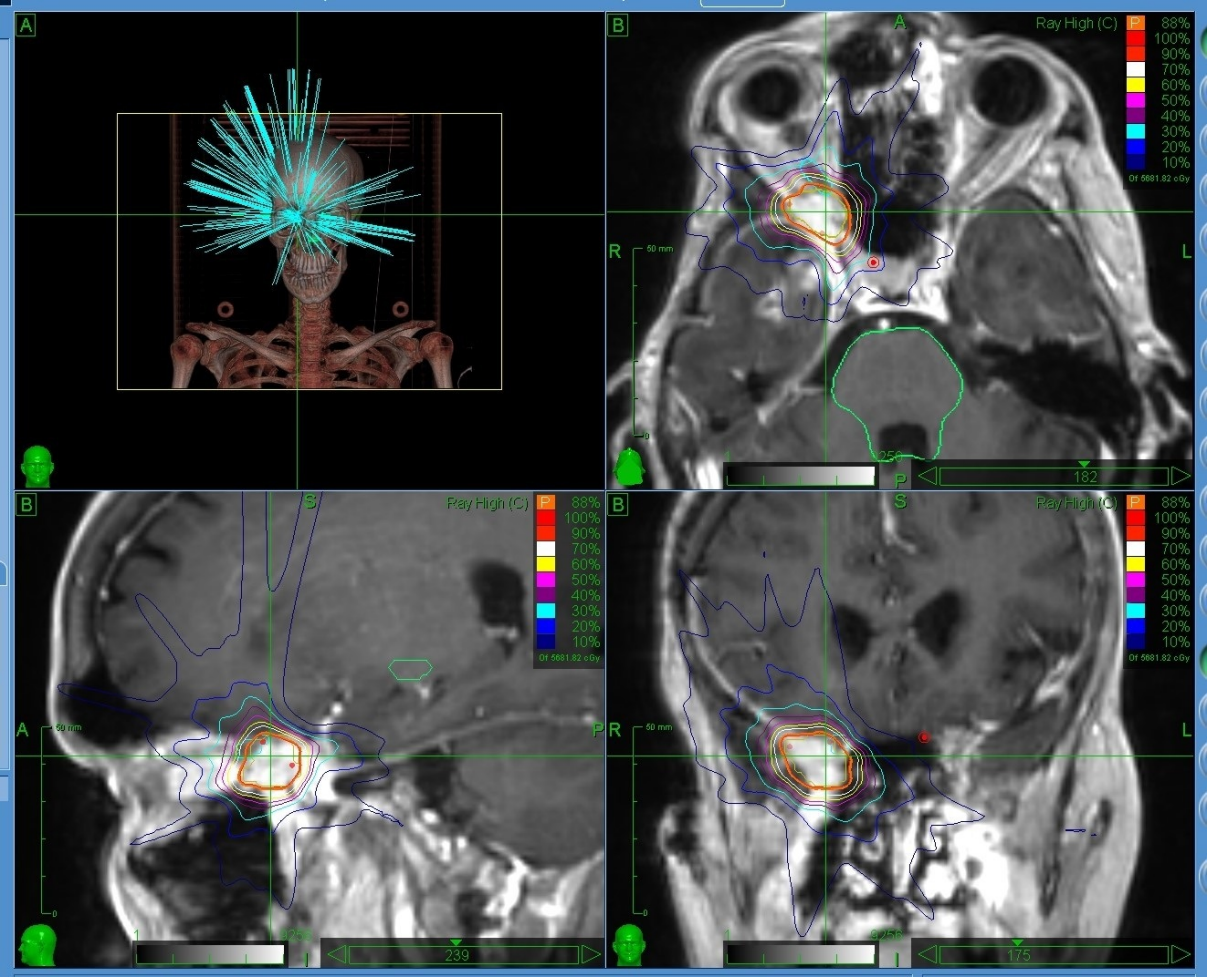
The intensity modulated radiation therapy treatment plan for the orbital metastasis.

**Figure 5:**
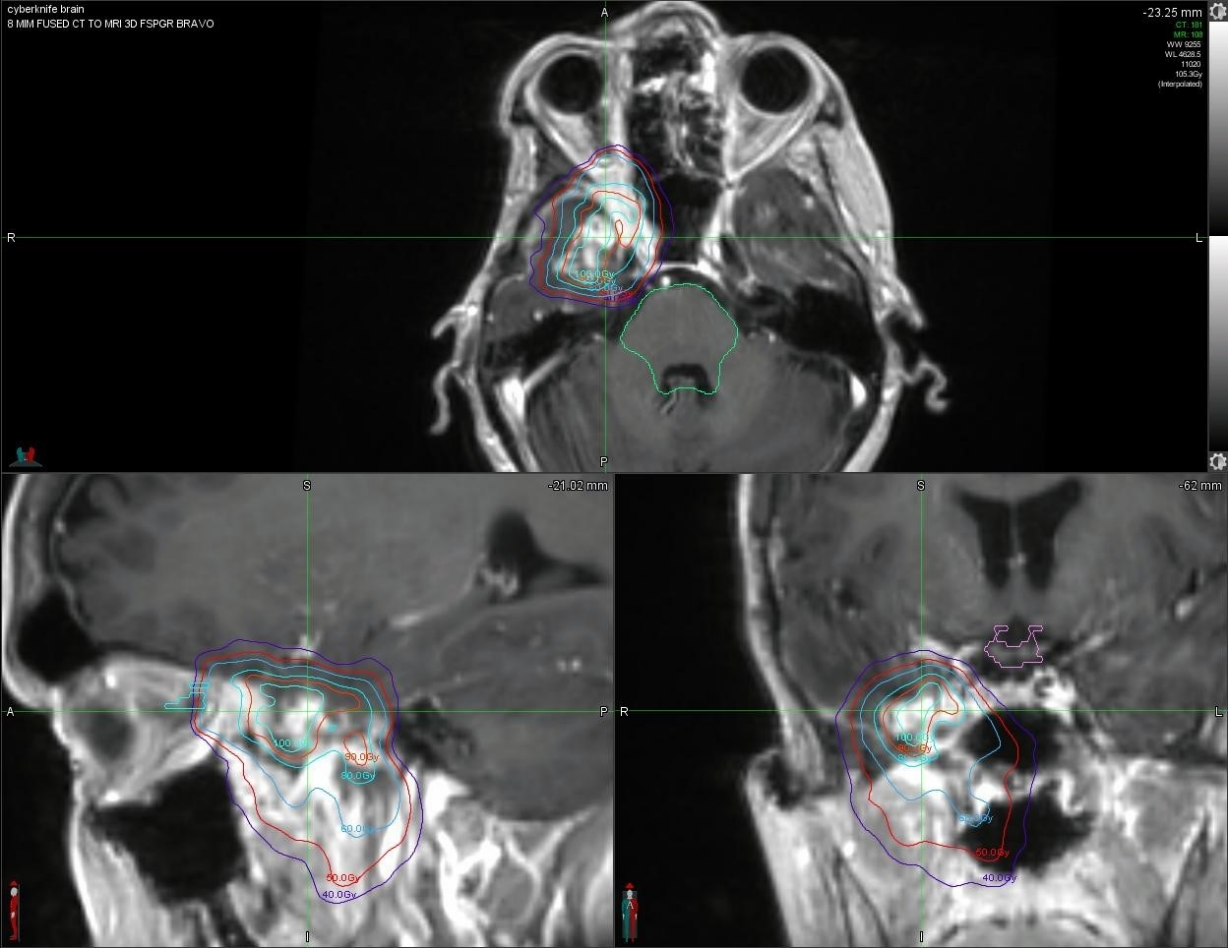
The computed tomography and the magnetic resonance imaging fusion of the orbital metastasis.

The patient was evaluated by the neurosurgery, ophthalmology, and neuro-ophthalmology at outside centers and no surgical intervention was recommended. The patient was started on dexamethasone 4 mg twice a day and received a third course of the radiation therapy on Cyberknife®. The dose delivered was 5000 cGy in 25 fractions of 200 cGy per day utilizing the intensity-modulated radiation therapy (IMRT) prescribed to the 80% isodose line, which covered 95% of the planning tumor volume (PTV) and 99% of the gross tumor volume (GTV) (Figure 4). The MIM ™ fusion (MIM Software, Cleveland, Ohio, United States of America) was performed for the previous treatments, and previous maximum cumulative doses to critical structures were reviewed; critical structures included the brainstem, optic chiasm, and the optic nerves (Figure 5). The patient completed the treatment in December 2016. At the time of completion, her vision was clinically restored and the ophthalmologic examination revealed OD of 20/30. She had been for follow-up in June 2017. The repeat MRI at that time showed stable disease and the ophthalmologic testing revealed 20/30 in the affected eye. As of November 2017, her visual recovery was stable.

Discussion

Review of the Metastatic ACC and the Current Treatment Strategies

The ACC has been relatively rare, yet locally aggressive cancer of the major and minor salivary glands [2]. It is characterized by an indolent course, with the high incidence of late local recurrence and distant metastases [2]. The most common sites of metastasis in ACC are the lungs, bone, and liver [4-5]; however, the intracranial metastases are not uncommon, with various studies reporting incidence from 4% to 22% [6]. This is most likely due to the tumor’s propensity for perineural invasion and spread along nerves [7]. Although metastases are common in this tumor, the ACC metastases to the orbit are exceedingly rare. To our knowledge, there has been only one other reported case of ACC metastases to the orbit [8]. The ACC primaries in the orbit are also rare, accounting for 1% of the orbital tumors [9]. These orbital tumors generally arise from the lacrimal gland tissue; however, there have been cases of the ACC primaries in the orbit that was not of lacrimal gland origin [9-13].

Currently, the mainstay of the treatment for primary ACC and local tumor recurrence consists of the radical excision with or without postoperative radiation using the external beam radiation therapy (EBRT) [5, 14]. There is some controversy in the literature regarding the role of the postoperative radiation in the treatment of ACC, but it is widely recommended due to the high incidence of recurrence [15]. When possible, the surgery and post-operative radiation continue to be the treatment of choice for metastases as well. With the surgery and adjuvant radiation, five-year survival is high, reported at between 64% and 95% [4-5, 15-17]. However, these numbers dwindle with time. A Danish study by Bjorndal, et al. reported a 15-year recurrence-free survival rate at 55% [4]. Another study performed by He, et al. reported a 10-year disease-free survival rate at 23% [15].

The surgical excision in this area was deemed to be risky due to the proximity of the tumor to the optic nerve. The alternatives to the surgery include the radiation therapy and chemotherapy. The role of chemotherapy in the management of the ACC metastases has been extensively studied, but there remains a lack of significant data regarding its usefulness [5]. In some studies, it has shown to be effective in treating symptoms of ACC metastases [18]. However, the chemotherapeutic agents to date have not shown any benefit in long-term survival rates for the metastatic ACC [18]. 

In terms of the radiation, common modalities include the EBRT, IMRT, volumetric modulated arc radiation therapy, stereotactic radiosurgery, particle radiation therapy, and brachytherapy. Of these modalities, the EBRT remains the mainstay of the treatment for the ACC [14]. Non-operative therapy with photon irradiation has a reported cure rate of 20% [19]. However, it has an increased incidence of local failure in comparison to combined modality treatment and is therefore generally reserved for the patients who are not surgical candidates or have unresectable tumors [19]. Ko, et al. reported three-year disease-free survival rates for individuals who received photon radiotherapy alone versus radiotherapy with surgery as 18.3% compared to 67.3%, respectively [16]. The subsequent disease-free survival rate for the radiotherapy alone group dropped to 9.2% at the five-year mark [16]. The IMRT is a form of EBRT that allows the radiation to be delivered at higher doses and with greater conformality as compared to the traditional EBRT [14]. This is especially beneficial when treating tumors located adjacent to radiosensitive structures, such as the orbit [14]. However, to our knowledge, the efficacy of the IMRT in relation to traditional EBRT for the treatment of ACC is yet to be studied. 

Particle irradiation has also been studied in treating salivary gland tumors. The fast neutron radiotherapy treatment of salivary gland tumors was compared to conventional photon and/or electron radiotherapy in terms of efficacy in the Radiation Therapy Oncology Group-Medical Research Council (RTOG-MRC) randomized trial [20]. The study found that fast neutron radiotherapy was superior to photon radiotherapy in local control rates: 67% for the neutron radiotherapy and only 17% for the photon radiotherapy [20]. However, there was no improvement in survival rates with neutron therapy, and the follow up final report of the RTOG-MRC revealed that neutron therapy resulted in more severe toxicity than photon therapy [20-21]. Takagi, et al. studied the efficacy of treating unresectable or the grade 4 ACC’s of the head and neck with the proton or carbon ion radiation. Five-year local control rates were 66% in unresectable tumors and 68% in grade 4 ACC’s [22]. However, similar to neutron therapy, 26% of the patients treated with protons or carbon ions experienced grade 3 or greater late toxicities [22]. In addition to unchanged survival rates and increased toxicity, there remains a paucity of particle therapy centers in the country, making access to particle therapies difficult for both the patients and practitioners.

Implications of this Case in the Intensity Modulated Radiation Therapy Treatment of the Adenoid Cystic Carcinoma and Orbital Tumors

In this case, the patient with a known history of ACC metastases of the cavernous sinus and right temporal region presented with an ACC metastasis to the right orbital apex causing compressive optic neuropathy. The surgical excision in this area was deemed to be risky due to the proximity of the tumor to the optic nerve. Based on the extent of this patient’s visual loss, its effect on the quality of life, and the previous response of her metastases to the radiation therapy, we treated the gross tumor with radiation in an attempt to control the tumor and salvage of her vision as much as possible. As opposed to the EBRT, conventional fractionated IMRT via Cyberknife ™ was used due to the proximity of the tumor to the optic nerves and the risk of toxicity to the other surrounding orbital structures. During the treatment, she experienced no adverse effects and since has experienced no late-term toxicity as a result of the treatment. As of November 2017, she has had complete recovery of the vision. We were unable to find similar cases of complete visual recovery following the radiation treatment of the ACC metastases to the orbit. Therefore, this case demonstrates the efficacy of the IMRT in the treatment of the ACC metastases.

In a broader sense, this case also shows the efficacy of the IMRT in the treatment of orbital apex tumors. The orbit is a complex space with critical structures, including the optic nerve. This provides a uniquely challenging situation for the medical practitioners. The treatment is often multidisciplinary, especially in the treatment of metastatic orbital tumors [23]. The surgery is a common option for the treatment of orbital tumors; however, it is often difficult and risky due to the complex anatomy and dense network of critical neurovascular structures held within the orbit [24]. As an alternative to surgery, the radiation is a valid treatment option for orbital tumors. The EBRT has long been a mainstay of the treatment for orbital tumors [25]. However, the dose to the surrounding structures within the orbit is generally the same as to the target tissue, leading to significant side effects associated with this modality, namely dry eye, eyelash loss, cataract, neovascular glaucoma, radiation retinopathy, and optic neuropathy [14, 25]. As previously stated, the IMRT is more conformal than conventional EBRT, thereby decreasing the radiation dose too close critical structures [25]. With the precision of IMRT, we were able to treat the orbit and achieve near complete recovery of vision with no side effects. We, therefore, present the IMRT as a superior modality to the traditional EBRT in the treatment of orbital tumors.

## Conclusions

In this study, we present a case of nasopharyngeal ACC primary metastasizing to the orbital apex, a location in which, to our knowledge, only one other study has reported metastatic involvement. In addition, we present a case of symptomatic relief of compressive optic neuropathy with the use of radiation therapy (using IMRT modality) without surgery. We contend that metastases to the orbit in individuals with the history of the ACC should be included in the differential diagnosis when presenting an orbital mass. In addition, we present IMRT as an effective palliative treatment for the ACC and orbital tumors as a whole.
